# A Novel Thin-Film Nanocomposite Nanofiltration Membrane by Incorporating 3D Hyperbranched Polymer Functionalized 2D Graphene Oxide

**DOI:** 10.3390/polym10111253

**Published:** 2018-11-12

**Authors:** Quanling Xie, Shishen Zhang, Hanjun Ma, Wenyao Shao, Xiao Gong, Zhuan Hong

**Affiliations:** 1 Engineering Research Center of Marine Biological Resource Comprehensive Utilization, SOA, The Third Institute of Oceanography of the State Oceanic Administration, Xiamen 361005, China; 20620151152271@stu.xmu.edu.cn (S.Z.); 20620171151152@stu.xmu.edu.cn (H.M.); 2 Department of Chemical and Biochemical Engineering, College of Chemistry and Chemical Engineering, Xiamen University, Xiamen 361005, China; 20420162201530@stu.xmu.edu.cn; 3 Fujian Collaborative Innovation Center for Exploitation and Utilization of Marine Biological Resources, Xiamen 361005, China

**Keywords:** nanofiltration, thin-film nanocomposite, graphene oxide, hyperbranched polyester, carboxylation

## Abstract

In order to develop a high-performance thin-film nanocomposite (TFN) nanofiltration (NF) membrane, the functionalized graphene-based nanomaterial (GO-HBE-COOH) was synthesized by combining two-dimensional graphene oxide (GO) with a three-dimensional hyperbranched polymer, which was used as the novel nanofiller and successfully embedded into the polypiperazine-amide (PPA) active layers on polysulfone (PSU) substrates via interfacial polymerization (IP) process. The resultant NF membranes were characterized using ATR-FTIR, SEM, and AFM, while their performance was evaluated in terms of water flux, salt rejection, antifouling ability, and chlorine resistance. The influence of GO-HBE-COOH concentration on the morphologies, properties, and performance of TFN NF membranes was investigated. With the addition of 60 ppm GO-HBE-COOH, the TFN-GHC-60 NF membrane exhibited the optimal water flux without a sacrifice of the salt rejection. It was found that the introduction of GO-HBE-COOH nanosheets favored the formation of a thinner and smoother nanocomposite active layer with an enhanced hydrophilicity and negative charge. As a result, TFN NF membranes demonstrated a superior permeaselectivity, antifouling ability, and chlorine resistance over the conventional PPA thin-film composite (TFC) membranes.

## 1. Introduction

Pressure-driven membrane separation technology with a selectively permeable membrane belongs to a kind of physical separation process, which has the advantages of high efficiency, energy saving ability, modular design, friendliness to the environment, and so on. Nanofiltration (NF) is the newest of the four classes of pressure-driven membranes whose pore size is between reverse osmosis (RO) and ultrafiltration (UF). To date, polyamide (PA) thin-film composite (TFC) NF is widely applied in many industries including water treatment, pharmaceutical and biotechnology, and food engineering due to its distinguishing characteristics such as low rejection to monovalent ions, high rejections to divalent ions and low Mw compounds, higher flux, and lower operation pressure [1]. Nevertheless, PA-TFC NF membrane still encounters great challenges such as a trade-off between permeability and selectivity, membrane fouling, chlorination, and concentration polarization [2]. In order to overcome these challenges, organic-inorganic hybrid membranes have been developed by combining the advantages of organic materials and inorganic materials. In recent years, the rapid development of nanomaterials plays an important role in promoting the development of novel composite membranes. Especially, the application of two-dimensional (2D) graphene nanomaterials, represented by graphene oxide (GO), has received increasing attention. Thin-film nanocomposite (TFN) NF membranes incorporating an appropriate amount of GO nanosheets into the top active layer and/or the porous support layer have demonstrated a superior performance over the traditional PA-TFC NF membranes [3,4,5,6,7,8,9,10]. Because the top PA active layer is believed to play a crucial role in the permeaselectivity and antifouling performance, most studies related to GO-modified TFN NF membranes focus on the optimization of the active layer rather than the porous support layer.

GO with abundant oxygen-containing functional groups is normally regarded as a kind of hydrophilic nanomaterial. Actually, the structure of GO has been clarified to be amphipathic with an edge-to-center distribution of hydrophilic and hydrophobic domains [11,12,13]. Therefore, the chemical functionalization of GO is needed to enhance the hydrophilicity, dispersibility as well as the compatibility with the polymer matrices. Our group used a small molecular modifier, maleic anhydride (MAH), reacted with the electrophilic groups of GO and synthesized MAH functionalized GO (MAH-GO) [14]. It was found that TFN NF membranes introducing MAH-GO into the active layer displayed a better performance than GO-embedded TFN NF membranes.

Hyperbranched polymers (HBPs) have attracted more and more interest in membrane preparation due to the three-dimensional (3D) spherical structure and unique physicochemical properties compared to their linear analogues [15,16,17]. Moreover, HBPs showed a good interfacial compatibility with cross-linked polyamide matrix and the intra-molecules nanovoids inside HBPs offered additional water channels to enhance membrane flux. The modified HBPs containing amino, alcohol, and carboxylic acid end groups were used for reducing protein adsorption by introducing a high density of hydrophilic functional groups at the membrane surface [18]. Many kinds of HBPs such as hyperbranched polyester (HPE) [17,19,20,21,22,23] and hyperbranched polyethyleneimine [24,25,26,27] have been explored to improve membrane performance. Kong et al. [20] introduced a small amount of HPE used as the macromonomer into the aqueous phase. They found that the HPE embedded in the active layer significantly increased the permeability without sacrificing the salt rejection for the prepared TFC NF membranes. Furthermore, HPE with a 3D spherical structure preferred to be deposited on the top surface of the substrate rather than snake through the pores like linear molecules [17].

In order to make use of 2D graphene-based nanomaterials and 3D HBPs in this study, GO was firstly modified with hydroxyl-terminated HPE to synthesize HPE functionalized GO (GO-HPE), and then GO-HPE was further reacted with succinic anhydride to obtain GO-HPE-COOH containing a large number of hydrophilic carboxyl groups. Finally, the resultant GO-HPE-COOH nanosheets used as novel nanofillers were added into the aqueous phase and incorporated into the polypiperazine-amide (PPA) active layer during the interfacial polymerization (IP) process. The effect of GO-HPE-COOH concentration on the morphologies, properties, and performance of the resulting TFN NF membranes were investigated in-depth. Furthermore, the influencing mechanisms of GO-HPE-COOH on the IP process and the formation of the nanocomposite active layer were discussed.

## 2. Experimental

### 2.1. Materials

Polysulfone (PSU, Ultrason^®^S 6010), PVP K30, and PVP K90 were produced by BASF (Ludwigshafen, Germany). Succinic anhydride, tetrahydrofuran (THF), dimethylformamide (DMF), dimethyl sulfoxide (DMSO), triethylamine (TEA), n-hexane, sodium hypochlorite, sodium sulfate, magnesium sulfate, sodium chloride, magnesium chloride, and hydrochloric acid (36–38%) were of analytical grade and produced by Sinopharm (Shanghai, China). SOCl_2_, piperazine (PIP), and trimesoyl chloride (TMC) were purchased from Aladdin (Shanghai, China). HBE was produced by HyPerBranched Polymers Co. Ltd (Wuhan, China). Bovine serum albumin (BSA) was purchased from BBI Life Sciences Corporation (Shanghai, China).

### 2.2. Preparation of GO and GO-HBE-COOH

**Figure 1:**
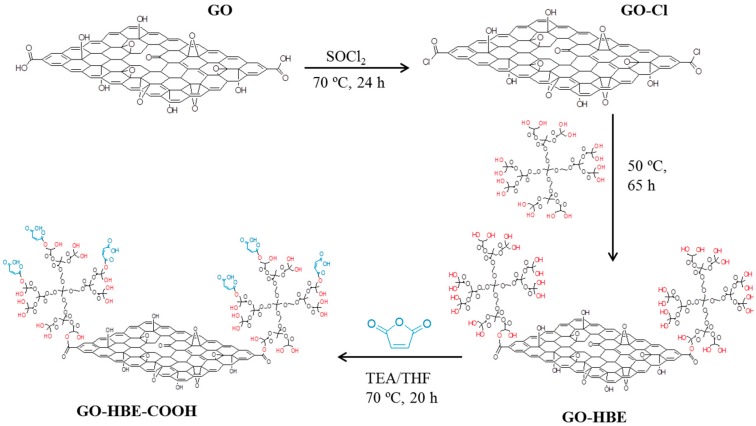
The schematic of the synthesis route of GO-HBE-COOH.

GO were obtained using the modified Hummer’s method [14]. The esterification procedure for GO was as follows (Figure 1): 100 mg of GO were reacted in 20 mL of SOCl_2_ (containing 1 mL DMF) at 70 °C for 24 h to convert the carboxylic acids into acyl chlorides (acyl chloride-derivative GO, GOCl). After centrifugation at 12,000 rpm for 10 min, the supernatant was decanted and the remaining solid was washed three times with anhydrous THF. The obtained GOCl solid was dried at room temperature under vacuum. A mixture of the resulting GOCl and 4.5 g HBE (45 mmol equivalent to the OH group) was dispersed in 100 mL DMSO and stirred vigorously at 50 °C for 65 h. After centrifugation, the remaining solid was washed three times with methanol. The obtained GO-HBE solid was dried at room temperature under vacuum. Then the carboxylation procedure for GO-HBE was carried out as follows: the obtained GO-HBE were dispersed in 60 mL THF. A total of 2.07 g of succinic anhydride and 1.0 mL of TEA were dissolved in 60 mL THF, which was mixed with GO-HBE suspension and condensate refluxed at 70 °C for 20 h. After centrifugation, the remaining solid was washed three times with THF. The obtained GO-HBE-COOH solid was dried at room temperature under vacuum.

### 2.3. Preparation of Composite NF Membrane

The structure and property of the porous support layer are also in close relation to the performance of the resultant composite NF membrane. During the preliminary study in our laboratory, the optimum PSU concentration and casting solvent for fabricating the porous supports was found to be 15.0 wt.% and NMP, which was consistent with the previous report [28]. GO-HBE-COOH nanofillers with various concentrations (0–80 ppm) was first dispersed into the 2.0 wt.% PIP aqueous solution containing 2.0 wt.% TEA as an acid acceptor by ultrasonic treatment for 40 min. Then 100 mL of the resultant aqueous solution was poured on the top of the PSU support and held for 2 min before removing the excess aqueous solution. Subsequently, 100 mL of an organic solution containing 0.1 *w*/*v* % TMC dissolved in n-hexane was poured on the top of the PIP saturated PSU support and drained off after 1 min contact time. Finally, the resulting composite membranes were heat cured at 60 °C for 8 min to enhance the IP reaction. The obtained nanocomposite membranes were named TFN-GHC-X, where X denoted the GO-HBE-COOH concentration (ppm) in the aqueous solution. The composite NF membrane without GO-HBE-COOH was named TFC-blank.

### 2.4. Characterization of GO and GO-HBE-COOH

The functional groups and chemical structure differences of GO and GO-HBE-COOH were characterized by Fourier transform infrared spectroscopy (FT-IR, Bruker VERTEX 70, Fällanden, Switzerland). Their morphologies were observed with a transmission electron microscopy (TEM, TALOS F200, Hillsboro, OR, USA) operated at 200 kV. The X-ray diffraction (XRD) with Cu Kα radiation (λ = 0.154 nm, D/max-rB 12 kW, Rigaku, Tokyo, Japan) was performed (with an emission current of 15 mA and the accelerating voltage of 35 kV) and the XRD data were processed by the MDI Jade 6.0 software.

### 2.5. Characterization of Composite NF Membranes

Attenuated total reflectance-Fourier transform infrared spectroscopy (ATR-FTIR, Bruker VERTEX 70, Fällanden, Switzerland) was employed to characterize the presence of GO-HBE-COOH in the fabricated TFN membranes. The membrane hydrophilicity was assessed by the static water contact angle using a contact angle goniometer (SPCAX3, HARKE, Beijing, China). The instantaneous contact angle was recorded after 30 s of equilibration time and at least five water contact angles were averaged to get a reliable value. The top membrane surfaces and their cross-sectional morphologies were observed using a scanning electron microscope (SEM, Sigma, Zeiss, Jena, Germany) operating at 15 kV. An atomic force microscope (AFM, MI5500, Agilent, Santa Clara, CA, USA) was used to characterize the morphology and roughness of the top membrane surface in tapping mode using the probe (OLTESPA-R3, Bruker, Billerica, MA, USA) with a spring constant of 2 N·m^−1^.

### 2.6. Membrane Performance

The NF filtration experiments were performed using a laboratory membrane separation device (FlowMem-0021-HP, Xiamen Filter and Membrane Technology, Xiamen, China). The composite membranes were pre-pressurized at 0.6 MPa and 25 ± 1 °C for 30 min before measuring their separation performance. Four representative salt solutions were adopted to evaluate the retention and selectivity. The diffusion coefficient and Stokes radius of various ions are listed in Table 1 [29]. The water flux and the rejection (*R*) were calculated using the Equations (1) and (2), respectively:

(1) $$J=\frac{V}{A∆t}$$

where *J* is the water flux (L·m^−2^·h^−1^), *V* is the permeate volume (L), *A* is the effective membrane area (m^2^), and Δ*t* is the filtration time (h).

(2) $$R\left(\%\right)=\left(1-\frac{C_{p}}{C_{c}}\right)\times 100\%$$

where *C_p_* and *C_c_* refer to the salt concentration in the permeate and in the concentrate, respectively.

The antifouling abilities of the TFC-blank and the selected TFN membranes were assessed using BSA as the model foulant. First, the operation of water filtration was running for 60 min at 0.6 MPa. Then the operation of the BSA filtration was carried out for another 60 min using a 2 g·L^−1^ BSA solution instead of pure water. After washing the fouled membranes with pure water, a complete cycle including water filtration and BSA filtration were repeated.

The chloride resistance of the TFC-blank and the selected TFN membranes was evaluated based on the variation of permeaselectivity. The tested membranes were immersed in a 2000 ppm NaClO solution with a neutral pH. In order to remove the residual NaClO solution before reassessing the membrane performance, the chlorinated membrane was rinsed thoroughly with pure water and soaked in pure water for at least 1 h.

## 3. Results and Discussion

### 3.1. Characterizations of GO and GO-HBE-COOH

**Figure 2:**
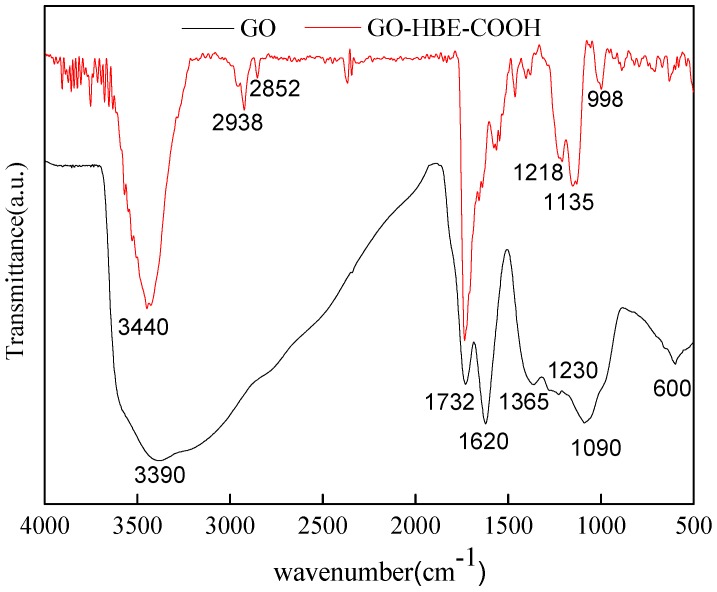
The FT-IR spectra of GO and GO-HBE-COOH.

The functional groups in GO and GO-HBE-COOH were analyzed via FT-IR (Figure 2). As reported in our previous studies [14,30], the GO spectrum displayed several typical characteristic peaks assigned to the oxygen-containing hydrophilic groups on the basal plane and edge: the broad band at 3390 cm^−1^ and the peak at 1365 cm^−1^ were assigned to the stretching vibration and deformation vibration of –OH; the peak at 1732 cm^−1^ was assigned to the C=O stretching vibration of carboxylic groups; two bands at 1230 and 1090 cm^−1^ were assigned to the C–O in epoxy groups and C–O in alkoxy groups, respectively. After the surface covalent modification with HBE and succinic anhydride, the broad band at 3390 cm^−1^ became sharp, and two new peaks appearing at 2938 cm^−1^ and 2852 cm^−1^ were assigned to the C–H stretching vibration from methyl and methylene. Furthermore, the peak intensity at 1732 cm^−1^ assigned to the C=O stretching vibration of carboxylic groups was significantly enhanced. All these above observations confirmed the successful synthesis of GO-HBE-COOH.

**Figure 3:**
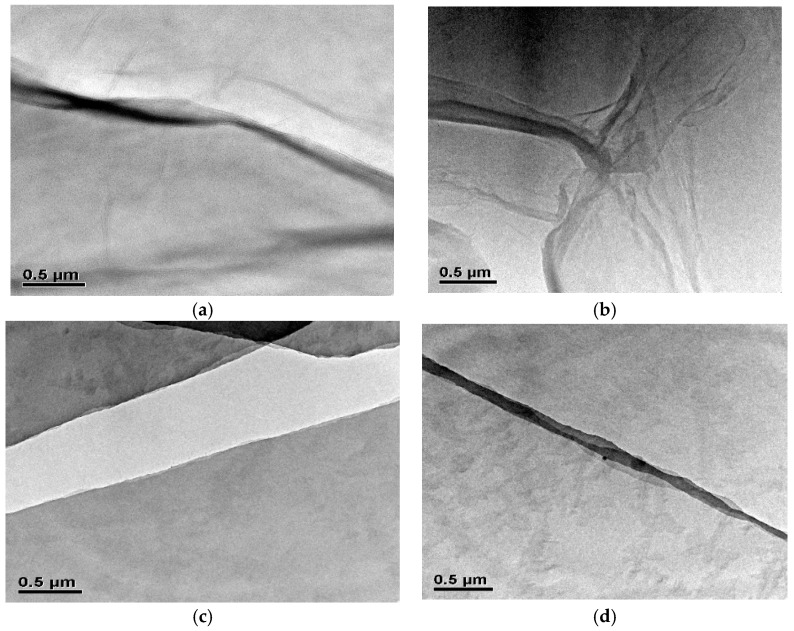
The TEM images of GO (**a**,**b**) and GO-HBE-COOH (**c**,**d**).

According to Figure 3, both GO and GO-HBE-COOH displayed one to several layers with some wrinkling within large flat areas, leading to the color being varied from transparent to semitransparent. Obviously, GO-HBE-COOH presented a similar lamellar structure and morphology as GO nanosheets. It was deduced that GO-HBE-COOH attaching hyperbranched polymer via covalent bonding did not alter the original structure and morphology of the GO nanosheets.

**Figure 4:**
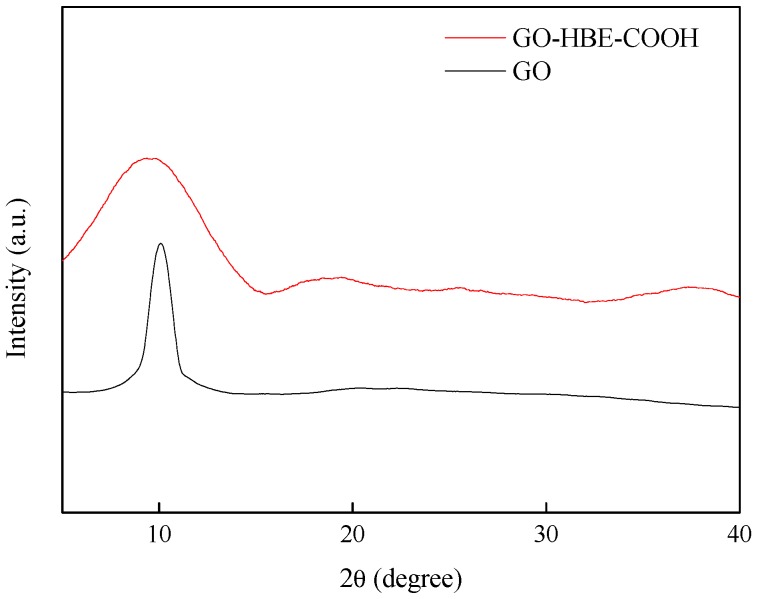
The XRD patterns of GO and GO-HBE-COOH.

**Figure 5:**
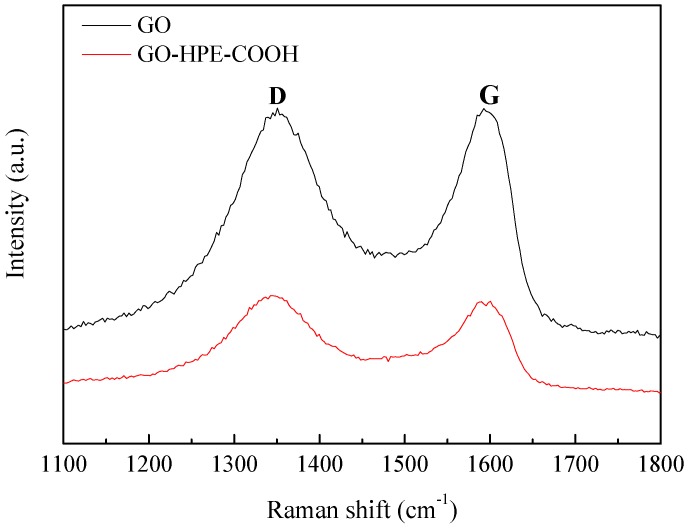
The Raman spectra of GO and GO-HBE-COOH.

The XRD patterns of GO and GO-HBE-COOH are shown in Figure 4. A sharp feature diffraction peak at 10.08° was attributed to the (002) plane of GO [31], which corresponded to a 0.877 nm d-space calculated from the Bragg equation. After introducing the hyperbranched polymer onto the basal plane of GO nanosheets, the feature diffraction peak broadened and left-shifted to 9.18°, corresponding to a 0.963 nm d-space. Because HBE was a kind of highly branched 3D spherical macromolecule, the introduction of a hyperbranched polymer weakened the interactions and increased the distance between graphene layers. This result was further confirmed by the Raman analysis. According to Figure 5, both GO and GO-HBE-COOH displayed two characteristic broad peaks at 1350 cm^−1^ (D band) and 1592 cm^−1^ (G band). D band was assigned to *sp^3^* hybridized carbons or the disordered graphite structure, and G band was attributed to *sp^2^* hybridized carbon atoms or the highly oriented graphite structure. Furthermore, the intensity ratio of D band to G band (*I_D_/I_G_*) in GO-HBE-COOH was slightly greater than that of GO, indicating an increase in surface defects after GO was modified with HBPs. It was concluded that HBPs was successfully grafted to the surfaces or edges of GO nanosheets.

### 3.2. Characterization of Composite NF Membranes

**Figure 6:**
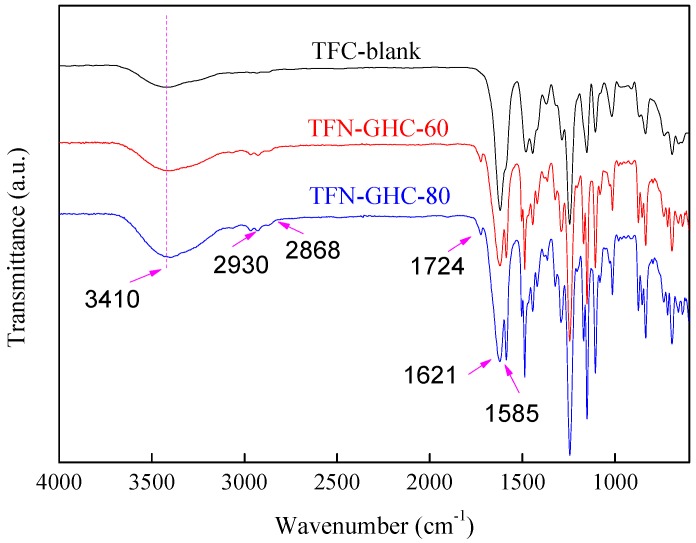
The ATR-FTIR spectra of composite NF membranes.

**Figure 7:**
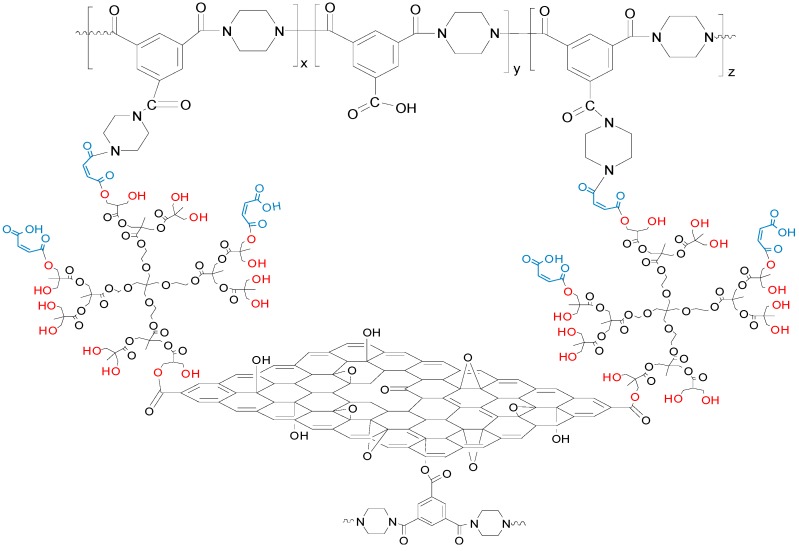
The schematic illustration of interactions between GO-HBE-COOH and PPA.

In order to elucidate the successful inclusion of GO-HBE-COOH nanosheets into the nanocomposite active layer, ATR-FTIR analysis was used to analyze the chemical groups on the surface of the membrane (Figure 6). The characteristic peaks of TFC-blank and TFN-GHC membranes at 1621, 1585, and 3410 cm^−1^ were attributed to the carbonyl stretching vibration of amide-I, the couplings of in-plane N–H bending, and the C–N stretching vibrations of amide II band, and the hydroxyl stretching vibration, respectively. This confirmed that the formation of PPA active layer on the top of PSU support after IP reaction. Compared to TFC-blank membrane, TFN-GHC membranes presented new absorption peaks at 2930, 2868, and 1724 cm^−1^, derived from the characteristic peaks of GO-HBE-COOH. Furthermore, these characteristic peaks and the broad band at 3410 cm^−1^ were enhanced with the increase of GO-HBE-COOH concentration. These results indicated that GO-HBE-COOH nanosheets were successfully embedded into the PPA active layer via IP reaction. Besides the hydrogen bonding between the oxygen-containing groups of GO-HBE-COOH and the PPA polymer, GO-HBE-COOH probably reacted with PIP and TMC monomers due to the abundant carboxyl groups. It was speculated that GO-HBE-COOH nanosheets were introduced into the active layer via covalent bindings (Figure 7), which ensured the long-term stability of GO-HBE-COOH nanosheets in the PPA matrix.

**Figure 8:**
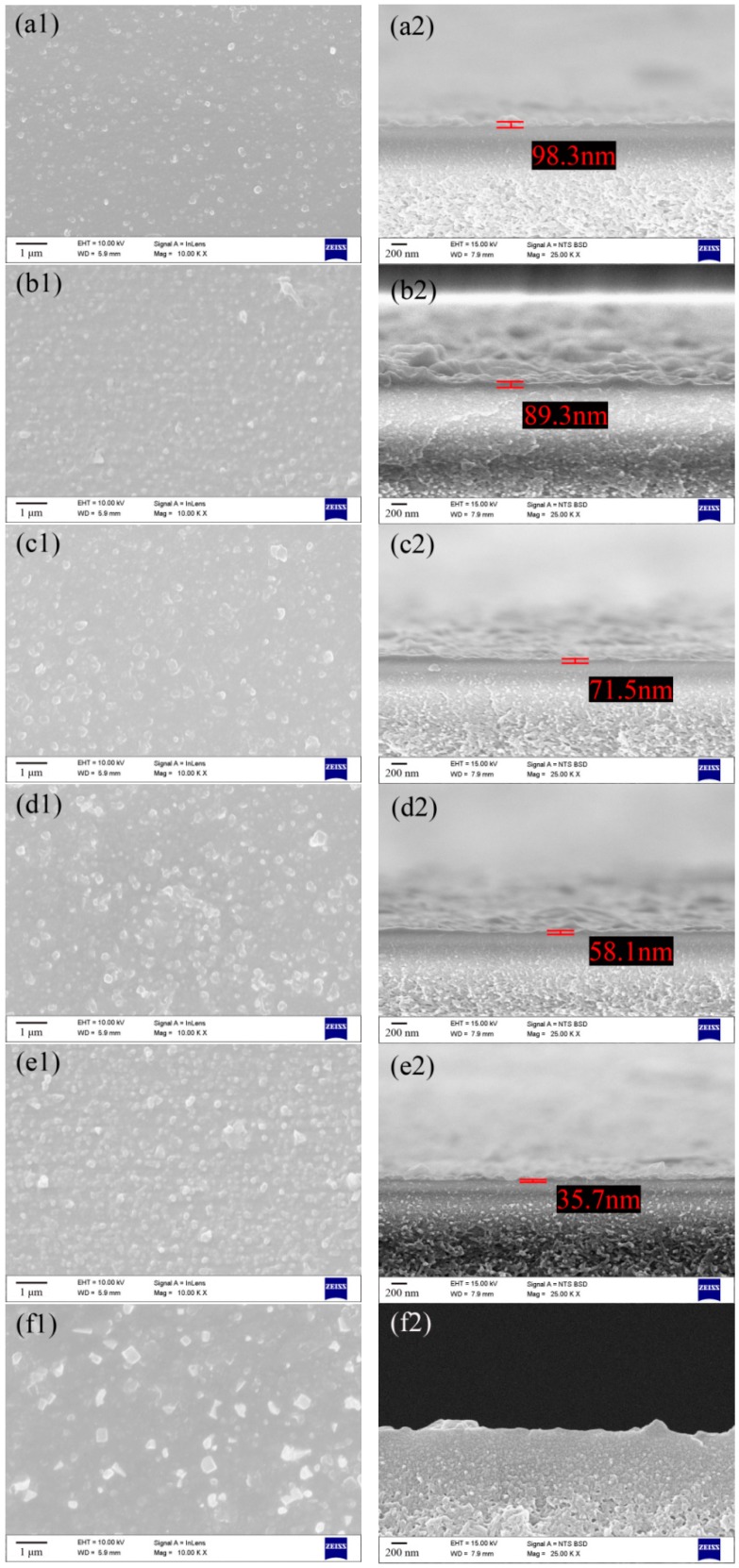
The SEM images of surfaces and cross-sections of composite NF membranes: TFC-blank (**a1**,**a2**), TFN-GHC-10 (**b1**,**b2**), TFN-GHC-20 (**c1**,**c2**), TFN-GHC-40 (**d1**,**d2**), TFN-GHC-60 (**e1**,**e2**), TFN-GHC-80 (**f1**,**f2**).

As shown in Figure 8, all the composite NF membranes exhibited typically composite structures consisting of an ultrathin active layer and a porous sublayer. Beside TFN-GHC-80 membrane, the other membranes exhibited the dense active layers with discrete nodular structures. When the GO-HBE-COOH concentration in the aqueous phase reached 80 ppm, some block shapes appeared on the top surface of the active layer which may be caused by the agglomeration of excessive GO-HBE-COOH nanosheets. Interfacial polymerization belongs to a reaction-diffusion process. The diffusion rate of the PIP monomer plays a crucial role in the formation of the PPA active layer. After introducing GO-HBE-COOH into the PIP aqueous phase, the steric hindrance effect arose from GO-HBE-COOH nanosheets and the hydrogen bonding between the PIP monomer and the abundant oxygen-containing hydrophilic groups of GO-HBE-COOH would slow down the PIP diffusion into the organic phase during the IP process [32]. Consequently, the amount of PIP monomers available for the IP reaction decreased within the same reaction time. Correspondingly, the thickness of the nanocomposite active layers obviously reduced with the increase of GO-HBE-COOH concentration. Notably, it seemed that the thickness of the nanocomposite active layer was too small to measure when the GO-HBE-COOH concentration was 80 ppm. In addition, the reduction in the thickness of the active layer was expected to reduce the membrane filtration resistance and increase the membrane permeability.

**Figure 9:**
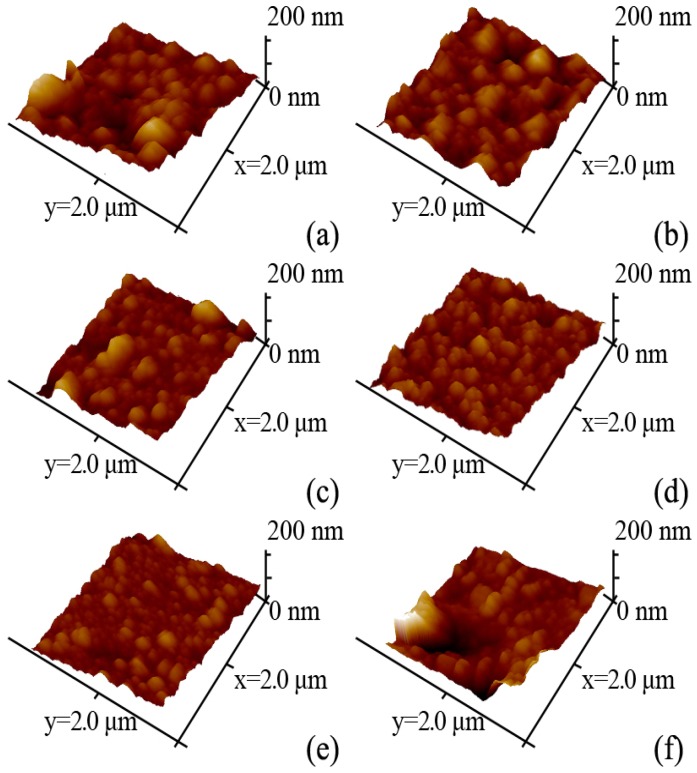
The three-dimensional AFM surface topography images of composite NF membranes: (**a**) TFC-blank; (**b**) TFN-GHC-10; (**c**) TFN-GHC-20; (**d**) TFN-GHC-40; (**e**) TFN-GHC-60; (**f**) TFN-GHC-80.

According to Figure 9 and Table 2, the surface roughness of the TFN-GHC membranes firstly decreased and then increased with an increase in the concentration of GO-HBE-COOH as compared with the TFC-blank membrane. As discussed above, the introduction of GO-HBE-COOH nanosheets slowed down the diffusion rate of the PIP monomer to the organic phase and accordingly hindered the formation of the PPA active layer, not only leading to a decrease of the active layer thickness but also to a reduction of the surface roughness. Nevertheless, when the GO-HBE-COOH concentration reached 80 ppm, due to the agglomeration of the excessive GO-HBE-COOH nanosheets, some block shapes appeared on the top surface of the active layer, resulting in an increase of the surface roughness.

**Figure 10:**
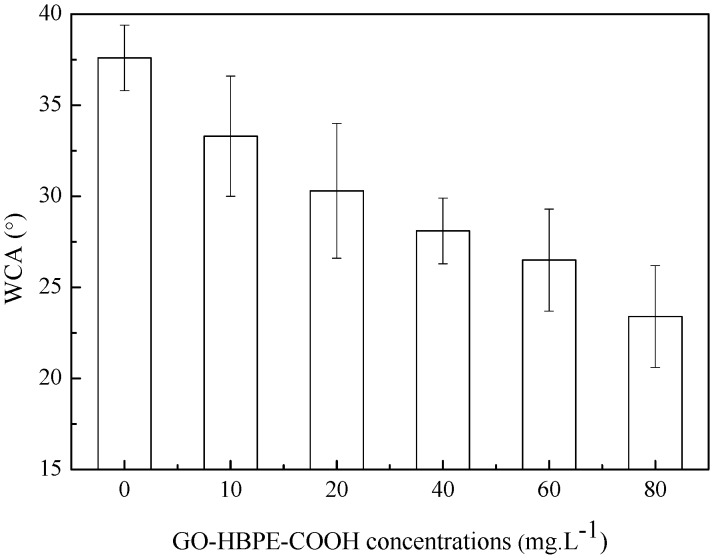
The effect of the GO-HBE-COOH concentration on WCA.

The water contact angle (WCA) of non-porous membranes such as NF and RO is mainly affected by the intrinsic wettability of the membrane material and the surface roughness. In general, the non-porous membrane having a rougher surface with the stronger hydrophilicity displays a smaller WCA. In this study, the variation of surface roughness was within 22 nm, so the influence of surface roughness on WCA could be negligible. As shown in Figure 10, the WCA of TFN-GHC membranes gradually decreased with the increase of GO-HBE-COOH concentration. This meant that TFN-GHC NF membranes became more and more hydrophilic with the increasing GO-HBE-COOH concentration. It was well understood that the enhanced hydrophilicity of TFN-GHC membranes was ascribed to the incorporated GO-HBE-COOH nanosheets having a large number of hydrophilic oxygen-containing groups, particularly an increasing amount of carboxyl groups.

### 3.3. Membrane Performance

**Figure 11:**
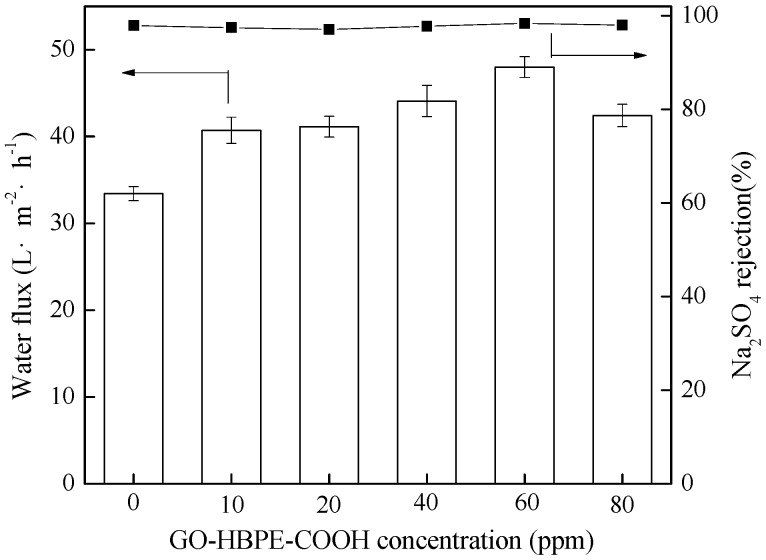
The water fluxes and rejections of composite NF membranes.

The effects of the GO-HBE-COOH concentration on the performance of composite NF membranes were evaluated according to the water flux and salt rejection (Figure 11). As discussed above, the nanocomposite active layer became thinner, smoother, and more hydrophilic with the addition of 10–60 ppm GO-HBE-COOH. As a result, the water fluxes of the corresponding TFN-GHC membranes gradually increased due to the reduction of membrane filtration resistance and a faster water flow on the membrane surface with less friction [30]. Additionally, the GO-HBE-COOH nanofiller having 2D nanosheets and a 3D spherical structure could increase the free volume between polymer chains and provided the additional water channels due to the nanometer-sized voids inside HBPs [20,33]. When the GO-HBE-COOH concentration reached the critical value of 60 ppm, TFN-GHC-60 membrane showed the maximum water flux of 48.0 L·m^−2^·h^−1^ equal to 143.7% of the TFC-blank membrane. More importantly, the TFN-GHC-60 NF membranes exhibited a high enhancement in water permeability while maintaining high rejection of salts, indicating that the introduction of an appropriate amount of GO-HBE-COOH nanosheets into the PPA active layer did not generate membrane defects. However, the water flux of TFN-GHC-80 membrane turned to decrease at the high concentration of 80 ppm because of the agglomeration of excessive GO-HBE-COOH nanofillers. Although the introduction of GO-HBE-COOH nanofillers in the aqueous phase resulted in the formation of a looser nanocomposite active layer which was adverse to the salt rejection, a large amount of ionizable and negatively charged groups in GO-HBE-COOH contributed to reducing the passage of salt due to the electrostatic repulsion effect. Consequently, TFN-GHC NF membranes retained a high salt rejection similar to the TFC-blank membrane.

**Figure 12:**
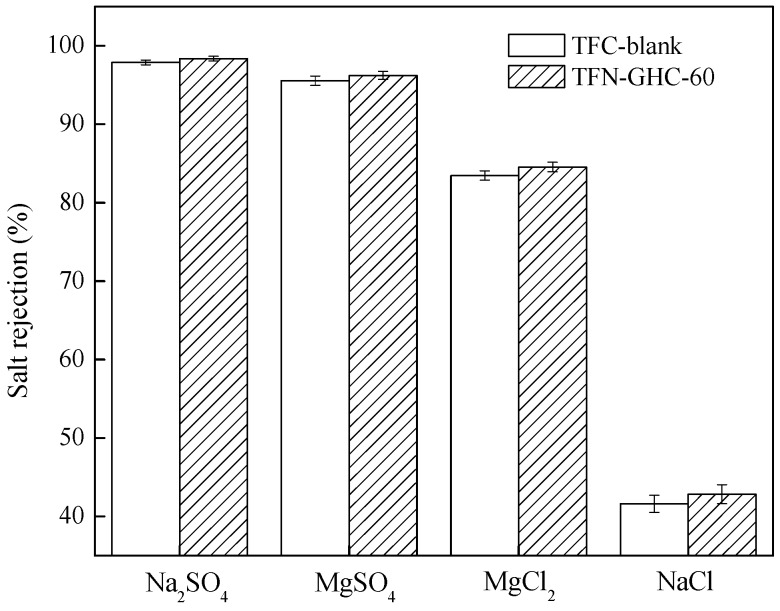
The four kinds of salt rejections of composite NF membranes.

It is known that the rejection of a charged NF membrane to an electrolyte is not only determined by the steric effect, but also depends on the electrostatic repulsion effect between the membrane and electrolyte. As shown in Figure 12, the salt rejections followed the order of Na_2_SO_4_ > MgSO_4_ > MgCl_2_ > NaCl, which was consistent with the separation characteristics of a negatively charged NF membrane. It was revealed that the co-ion played an important role in the rejection and transport rather than the counter-ion [34]. The negatively charged NF membrane would generate a stronger repulsive force to a divalent co-ion (SO_4_^2−^) than to a monovalent co-ion (Cl^−^) due to the electrostatic repulsion effect. Furthermore, the SO_4_^2−^ ion exhibits a lower diffusion coefficient and a larger Stokes radius than Cl^−^ ion (Table 1). Therefore, it was more difficult for the SO_4_^2−^ ion to pass through the active layer in comparison with Cl^−^ ion. Meanwhile, the negatively charged NF membrane had a stronger attraction for a divalent counter-ion (Mg^2+^) than a monovalent counter-ion (Na^+^). Consequently, the negatively charged NF membrane favored the rejection of the Na^+^ ion rather than the Mg^2+^ ion, resulting in a higher rejection of Na_2_SO_4_ than MgSO_4_. For the salt rejection having the same monovalent co-ion (Cl^−^), the higher rejection to MgCl_2_ than to NaCl was presumably due to the larger steric hindrance of the Mg^2+^ ion than to the Na^+^ ion.

**Figure 13:**
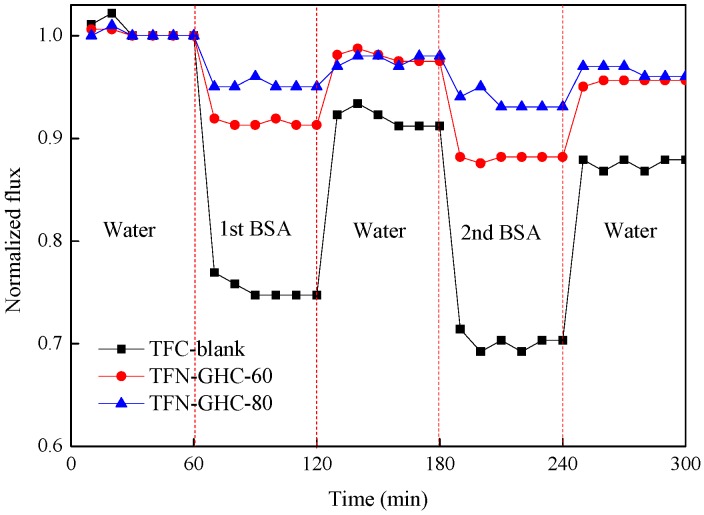
The time-dependent normalized flux of composite NF membranes.

Fouling tests were conducted on the TFC-blank, TFN-GHC-60, and TFN-GHC-80 membranes using BSA as the protein foulant. As shown in Figure 13, compared with the TFC-blank membrane, both the TFN-GHC NF membrane displayed a slower normalized flux decline, and the normalized flux decreased slowly with the increase of GO-HBE-COOH concentration. At the end of the second cycle of BSA filtration, the normalized flux of TFN-GHC-80 and TFN-GHC-60 membrane decreased by 7.0% and 12.0%, respectively, which was remarkably less than that of the TFC-blank membrane (30.0%). These results indicated that the TFN-GHC NF membranes were more resistant to protein contamination after the addition of GO-HBE-COOH nanosheets to the active layer. The antifouling abilities are typically affected by many membrane characteristics including hydrophilicity, surface roughness, and surface charge. Generally, a smooth membrane surface having a relatively strong hydrophilicity and a high surface charge exhibits a better antifouling ability. After the covalent binding of GO-HBE-COOH in the top active layer, TFN-GHC NF membranes showed a smoother surface with the enhancement in the membrane hydrophilicity and surface charge density, which promoted the formation of a regular hydration layer on the hydrophilic surface via hydrogen bonding, as well as strengthening the electrostatic repulsion between the negatively charged membrane surface and BSA. This would greatly reduce the BSA adsorption on the nanocomposite surface of the TFN-GHC NF membranes.

**Figure 14:**
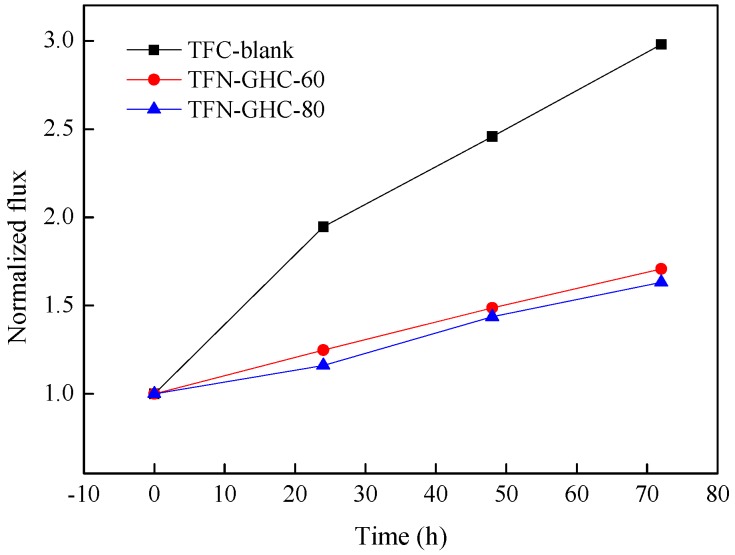
The effect of the chlorination exposure time on the water fluxes of composite NF membranes.

**Figure 15:**
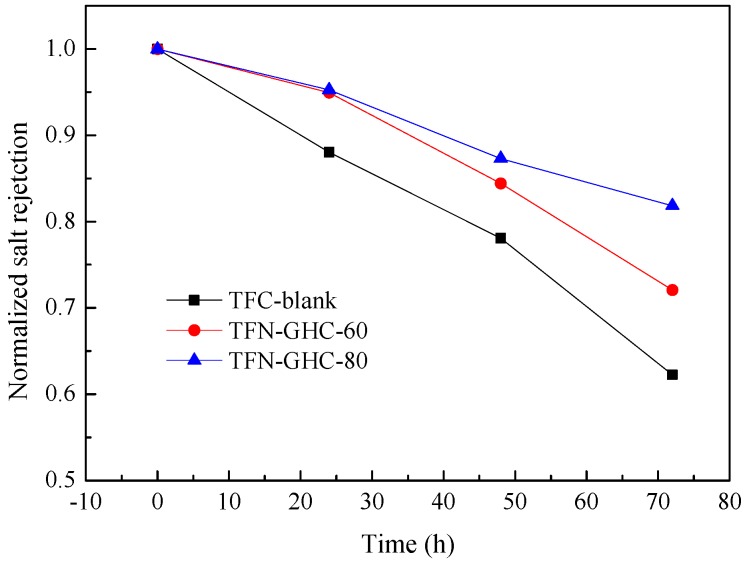
The effect of the chlorination exposure time on the salt rejections of composite NF membranes.

In view of the chlorinated degradation mechanism, the degradation of polyamide membranes normally occurs via a nucleophilic substitution reaction between chlorine and the hydrogen of the secondary amide group (−NH) [35]. So the secondary amines of PIP reacted with TMC forms the tertiary amines, which enhances the chlorine tolerance of PPA membrane. However, chlorinated degradation still occurred at the low abundance noncross-linked nitrogen atoms of PPA membrane [36]. To assess the chlorine resistance of TFC-blank and TFN-GHC NF membranes, the permeaselectivity was measured before and after the chlorine treatment. As shown in Figure 14 and Figure 15, all composite NF membranes displayed a reduction in salt rejection and an enlargement in water flux after chlorination, but the TFN-GHC membranes embedded with GO-HBE-COOH into the active layer showed an obvious inhibiting effect in water flux and salt rejection variations. After 72 h of chlorination exposure, the normalized salt rejections of the TFN-GHC-80 and TFN-GHC-60 membranes reduced to 81.8% and 72.0%, respectively, but the TFC-blank membrane reduced to 62.2%, which indicated that the chlorine resistance of the TFN-GHC NF membranes was enhanced with the increasing GO-HBE-COOH concentration. It was found that GO in the polymer matrix could trap the radicals because they had phenolic moieties with the radical scavenging ability [37]. Therefore, GO could absorb the chlorine radical to form O–Cl leading to a decrease in chlorine radical attacking the polyamide layer [38]. Because GO-HBE-COOH kept the skeleton structure of GO after modification with the hyperbranched polymer, it was deduced that GO-HBE-COOH could increase the chlorine resistance of the PPA-based membranes due to the formation of O–Cl during the chlorine radical attack. In addition, GO-HBE-COOH provided additional protections due to the huge specific surface area and the hydrogen bonding between PPA and GO-HBE-COOH [39].

### 3.4. Mechanisms

**Figure 16:**
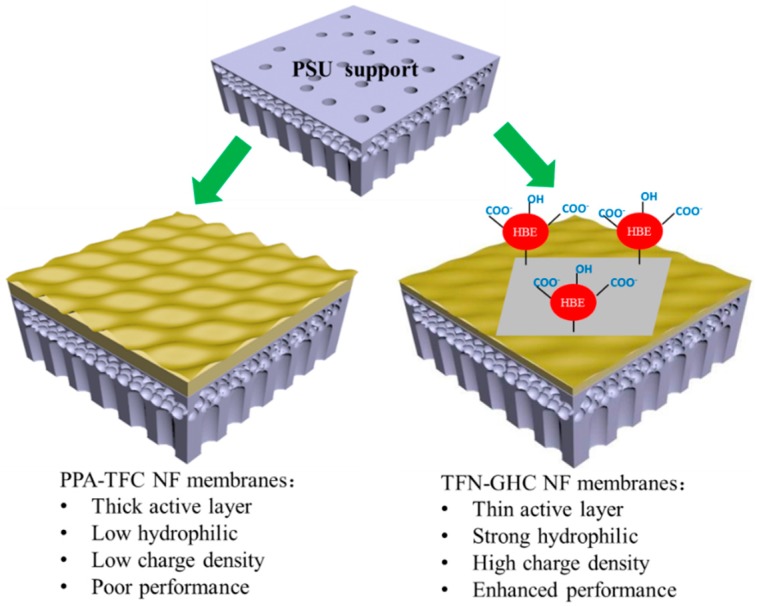
The schematic representation of speculated active layer formation.

It was well known that the growth rate of the polyamide layer is limited by the diffusion of the aqueous-phase monomer through the newly-formed polyamide [40]. That is, the diffusivity of amine monomer during the IP reaction significantly affects the properties, and performance of the fabricated TFC membrane. In this study, using the porous PSU supports, the introduction of GO-HBE-COOH nanosheets into the PIP aqueous phase could effectively hinder the diffusion rate of PIP monomers into the organic phase due to the steric hindrance effect of GO-HBE-COOH nanosheets and the hydrogen bonding between the PIP molecules and the oxygen-containing hydrophilic groups of GO-HBE-COOH nanosheets. At the same time, the synergistic combination of 3D hyperbranched macromolecules in GO-HBE-COOH nanosheets increased the free volume between the PPA chains and provided additional channels. Therefore, in comparison with the TFC-blank membrane, the TFN-GHC NF membrane exhibited a thinner, looser, and smoother nanocomposite active layer which resulted in reducing the membrane filtration resistance and obviously increasing the membrane permeability. Furthermore, the TFN-GHC NF membrane showed an enhanced hydrophilic and negatively charged surface due to a large number of hydrophilic and ionizable groups from GO-HBE-COOH nanosheets. As a result, TFN-GHC NF membranes embedded with GO-HBE-COOH nanosheets demonstrated a superior permeaselectivity, antifouling ability, and chlorine resistance over the traditional PPA-TFC NF membranes (Figure 16).

## 4. Conclusions

GO was firstly modified by 3D hyperbranched polyester and then carboxylated by succinic anhydride, and the resultant GO-HBE-COOH nanofiller was successfully incorporated into the PPA active layer via the IP process. The influences of the GO-HBE-COOH concentration on the morphologies, properties, and performance of the fabricated TFN NF membranes were investigated. With the addition of 60 ppm GO-HBE-COOH in the aqueous phase, the TFN-GHC-60 NF membrane exhibited the optimal water flux without a sacrifice of the salt rejection. Such a significant increase of the water flux resulted from the formation of a thinner, looser, and smoother nanocomposite active layer with more hydrophilicity, which was ascribed to the diffusion rate of PIP monomers hindered by the introduction of GO-HBE-COOH nanosheets. Furthermore, TFN-GHC NF membranes demonstrated a better antifouling ability and chlorine resistance with the increase of GO-HBE-COOH concentration. It was found that the high increase of antifouling performance was attributed to the smoother surface with the enhancement in the hydrophilicity and the negative charge, and the improved chlorine resistance was due to the protection of the PPA active layer provided by GO-HBE-COOH nanosheets. Therefore, we could use GO-HBE-COOH as a novel nanofiller incorporated into the active layer to develop the high-performance TFN NF membrane.

## Figures and Tables

**Table 1:** The parameters of relevant ions.

Ions	*z*	*D* (10^−3^ mm^2^·s^−1^)	*r*_s_ (nm)
Na^+^	1	1.333	0.183
Mg^+^	2	0.706	0.345
Cl^−^	−1	2.032	0.120
SO_4_^2−^	−2	1.065	0.229

Note: *z*, *D* and *r*_s_ refer to the charge number, diffusion coefficient and Stokes radius.

**Table 2:** The roughness of composite NF membranes.

Membrane ID	Roughness		
	*Ra* (nm)	*Rq* (nm)	*Rz* (nm)
TFC-blank	23.2	30.9	90.9
TFN-GHC-10	21.9	28.0	83.1
TFN-GHC-20	19.5	26.6	91.2
TFN-GHC-40	16.0	20.0	69.4
TFN-GHC-60	13.8	17.9	54.1
TFN-GHC-80	26.7	39.8	93.5
